# Sutureless and Rapid Deployment vs. Transcatheter Valves for Aortic Stenosis in Low-Risk Patients: Mid-Term Results

**DOI:** 10.3390/jcm12124045

**Published:** 2023-06-14

**Authors:** Claudio Muneretto, Lorenzo Di Bacco, Marco Di Eusanio, Thierry Folliguet, Fabrizio Rosati, Michele D’Alonzo, Diego Cugola, Salvatore Curello, Camila Mayorga Palacios, Massimo Baudo, Francesco Pollari, Theodor Fischlein

**Affiliations:** 1 Division of Cardiac Surgery, University of Brescia Medical School, 250123 Brescia, Italy; claudio.muneretto@unibs.it (C.M.); rosati.fabri@gmail.com (F.R.); dalonzomi@gmail.com (M.D.); massimo.baudo@icloud.com (M.B.); 2 Cardiac Surgery Unit, Ospedali Riuniti, 60126 Ancona, Italy; marco.dieusanio@ospedaliriuniti.marche.it; 3 Service de Chirurgie Thoracique et Cardio-Vasculaire, Hôpital H. Mondor, 94010 Créteil, France; thierry.folliguet@aphp.fr; 4 Cardiac Surgery Unit, ASST Papa Giovanni XXIII, 24127 Bergamo, Italy; dcugola@asst-pg23.it; 5 Cardiac Catheterization Laboratory, Spedali Civili, 250123 Brescia, Italy; scurello@libero.it; 6 Center for Neuroscience, Queen’s University, Kingston, ON K7L 3N6, Canada; 15mcmp@queensu.ca; 7 Cardiac Surgery Department, Klinikum Nürnberg-Paracelsus Medical University, 90419 Nürnberg, Germany; fpollari@gmail.com (F.P.); theodor.fischlein@klinikum-nuernberg.de (T.F.)

**Keywords:** Transcatheter Aortic Valve Implantation, Sutureless Aortic Valve, rapid deployment valves, permanent pacemaker implantation, para-valvular leaks

## Abstract

Background: Recent trials showed that TAVI is neither inferior nor superior to surgical aortic valve replacement. The aim of this study was to evaluate the outcomes of Sutureless and Rapid Deployment Valves (SuRD-AVR) when compared to TAVI in low surgical risk patients with isolated aortic stenosis. Methods: Data from five European Centers were retrospectively collected. We included 1306 consecutive patients at low surgical risk (EUROSCORE II < 4) who underwent aortic valve replacement by means of SuRD-AVR (n = 636) or TAVI (n = 670) from 2014 to 2019. A 1:1 nearest-neighbor propensity-score was performed, and two balanced groups of 346 patients each were obtained. The primary endpoints of the study were: 30-day mortality and 5-year overall survival. The secondary endpoint was 5-year survival freedom from major adverse cardiovascular and cerebrovascular events (MACCEs). Results: Thirty-day mortality was similar between the two groups (SuRD-AVR:1.7%, TAVI:2.0%, *p* = 0.779), while the TAVI group showed a significantly lower 5-year overall survival and survival freedom from MACCEs (5-year matched overall survival: SuRD-AVR: 78.5%, TAVI: 62.9%, *p* = 0.039; 5-year matched freedom from MACCEs: SuRD-AVR: 64.6%, TAVI: 48.7%, *p* = 0.004). The incidence of postoperative permanent pacemaker implantation (PPI) and paravalvular leak grade ≥ 2 (PVL) were higher in the TAVI group. Multivariate Cox Regression analysis identified PPI as an independent predictor for mortality. Conclusions: TAVI patients had a significantly lower five-year survival and survival freedom from MACCEs with a higher rate of PPI and PVL ≥ 2 when compared to SuRD-AVR.

## 1. Introduction

Transcatheter Aortic Valve Implantation (TAVI) represents, along with surgical aortic valve replacement (sAVR), a well-established treatment for severe aortic stenosis (AS) [1,2,3]. According to the latest guidelines, indications for TAVI include patients that are low-risk and younger than 75 years of age [4,5].

The safety and efficacy of TAVI in this subset of patients are supported by funded randomized controlled trials (RCTs), namely PARTNER 3, Evolut Low Risk and NOTION trial [3,6,7]. In these studies, short/mid-term outcomes have been shown to be comparable or even superior in the TAVI group when compared to sAVR [3,6,7]. However, long-term survival and valve durability data in this patient population are lacking. “Real-world” studies conversely showed an increased mortality rate when TAVI was compared to sAVR beyond the 1-year follow-up [2,8,9] in the same subset of patients. In particular, the OBSERVANT study reports five-year outcomes of low-risk patients treated by means of surgical aortic bioprostheses and transfemoral TAVI (TF-TAVI). In this prospective observational study, TAVI is associated with an increased incidence of all-cause death and MACCE at follow-up [2]. These findings suggest that procedural TAVI-related complications, such as paravalvular leaks (PVL) and a permanent pacemaker implant (PPI), could affect mid- and long-term survival [10,11]. Indeed, recent studies identified PPI and PVL following TAVI as independent predictors of mortality [11,12].

Sutureless and Rapid deployment valves (SuRD-AVR) have demonstrated a good safety profile associated with a significant reduction of cross-clamp and cardiopulmonary bypass times [13,14]. In addition, the rapid implantation technique facilitates sAVR in a minimally invasive approach, giving an additional advantage in terms of fewer blood transfusions and shorter hospital stay [15].

Recently, a meta-analysis reporting outcomes of SuRD compared to TAVI, including studies on low-intermediate risk profile patients, addressed worsening in the outcome of transcatheter valves over time in favor of surgery [16]. However, to date, the use of SuRD-AVR in RCTs has not been investigated in comparison with TAVI, especially in low surgical risk patients.

This European multi-institutional study sought to investigate the mid-term outcomes of patients with isolated aortic stenosis and low-risk profile treated with Su/RD-AVR versus TAVI.

## 2. Materials and Methods

This study is designed as a retrospective case-control study. The analysis enrolled 1306 consecutive low surgical risk patients treated in five European centers for isolated aortic stenosis from January 2014 to December 2019. Patients enrolled during the study period underwent aortic valve replacement by means of SuRD-AVR (636 patients) or TAVI (670 patients). Inclusion criteria were diagnosis of isolated aortic valve stenosis and a EuroSCORE II score ≤ 4%. Patients with bicuspid aortic valve Sievers type 0, with previous aortic valve replacement (either surgical AVR or TAVI) and patients needing any associated surgical or percutaneous interventions were excluded from the present analysis. The therapeutic strategy for each patient (Surgery vs. TAVI) was defined following a discussion with the local multidisciplinary Heart Team composed of cardiologists, cardiac surgeons, anesthesiologists, and other physicians involved in patients’ preoperative assessment according to the current guidelines at the time of the procedure. Survival data were obtained from either in-house information, telephone follow-up of patients, referring physicians or by contacting civil registries. Data from follow-ups are reported at the latest follow-up available. Patients provided informed consent to the procedure and data acquisition.

### 2.1. Sutureless and Rapid Deployment Aortic Valves (SuRD-AVR)

Surgical aortic valve replacement was performed either by means of a sutureless valve (Perceval S—Corcym srl, Saluggia, Italy) or by means of a rapid deployment valve (Intuity—Edwards Lifesciences, Irvine, CA, USA). Surgical techniques have been previously described in [17,18]. The surgical approach in 230 patients (36.2%) was performed by means of sternotomy, while a minimally invasive approach, by means of a “J shaped” ministernotomy, was performed in 406 patients (63.8%). Perceval S and Intuity valves were implanted in 392 (61.6%) and 244 (38.4%) patients, respectively. Intraoperative transesophageal echocardiography was routinely used in order to assess proper valve positioning and functioning at the end of each surgical procedure.

### 2.2. Transcatheter Aortic Valve Implantation (TAVI)

TAVI was performed via transfemoral (TF) approach in the majority of patients (TF: 569/670, 84.9%), followed by trans-subclavian (87/670, 13.0%) and trans-apical approaches (14/670, 2.1%). Implanted devices included either balloon expandable or self-expandable valves. In particular, 50.5% of patients (338/670) received the SAPIEN XT/SAPIEN 3 balloon-expandable valves (Edwards Lifesciences, Irvine, CA, USA); the Evolut R end Evolut Pro self-expandable valves (Medtronic, Minneapolis, MN, USA) were implanted in 37.2% of patients (249/670) while the Accurate Neo self-expandable valve (Symetis, Lausanne, Switzerland) and other devices (St Jude Portico, Lotus valve, Direct Flow Valve) were implanted in 7.2% (48/670) and 5.2% (35/670) of patients, respectively. Intraprocedural echocardiography and hemodynamic gradients were used to assess valve functioning and proper positioning.

### 2.3. Statistical Analysis

**Figure 1:**
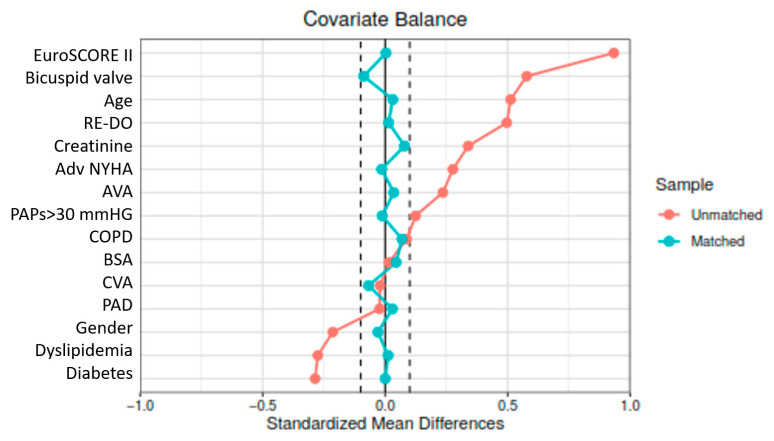
Love plot for Standardized mean differences pre- and post-propensity score matching.

Normal distribution was analyzed using the Kolmogorov-Smirnov test. Continuous variables were compared using an independent Student *t*-test with a two-tailed distribution if normally distributed. For non-normally distributed variables, the Mann-Whitney U-test was used. Categorical variables were compared using Chi-square χ^2^ or Fisher exact test, as needed. To balance baseline characteristics between groups, propensity score matching (PSM) was performed with a ratio of 1:1 using the nearest-neighbor method, without replacement and with 0.06 caliper. The matched standardized differences of each covariate in the matched cohorts were less than 10% (Figure 1), and the area under the receiver operating characteristic curve was 0.79. Preoperative characteristics are listed in detail in Table 1. Survival differences between the two groups were represented and compared using the Kaplan–Meier method and log-rank tests. Landmark analysis was performed to evaluate outcomes at different follow-up time points. The univariable and multivariable Cox proportional hazard regression models were used to investigate the effect of postoperative variables on all-cause mortality. A *p*-value ≤ 0.05 was considered statistically significant. The analysis was performed using SPSS (Version 22, IBM, Armonk, NY, USA).

### 2.4. Study Endpoints and Definitions

Patient and prosthesis outcomes were defined according to EACTS/ESC/EAPCI guidelines for reporting mortality and morbidity after cardiac surgery [19] and according to the Valve Academic Research Consortium 3 criteria [20].

Frailty was defined as the presence of two or more of the following criteria: 5 m walk-test time of more than 6 s, serum albumin level of less than 3.5 g/dL, and Katz Activities of Daily Living total score of 4 or less.

Patients were followed by clinical evaluation, including at least one physical examination. Follow-ups were 100% complete, and data were collected from the electronic medical record system of each institution.

The primary endpoints of the study were: (1) 30-day mortality; (2) overall survival (OS). The secondary endpoint was survival freedom from major adverse cardiovascular and cerebrovascular events (MACCEs) defined as death from all causes, stroke/TIA, endocarditis, reoperation, permanent pacemaker implantation (PPI) and paravalvular leak (PVL) of grade ≥ 2.

## 3. Results

### 3.1. Operative Outcomes

Procedural success (defined as per VARC-3 criteria) was comparable between the two groups (SuRD-AVR: 630 patients, 99.0% vs. TAVI: 655 patients, 97.7%, *p*-value = 0.062). Device failure in SuRD-AVR occurred in six patients; four cases occurred following sutureless valve implantation (one valve infolding, two paravalvular leaks > 2 and one annular rupture), while two occurred following Rapid Deployment Valve implantation (one paravalvular leak of grade > 2 and one iatrogenic membranous interventricular defect). In all these cases, a conventional sutured aortic valve was then implanted. Among the six patients with device implantation failure undergoing aortic valve re-implantation, two patients died perioperatively.

Device failure in the TAVI group occurred in 15 patients. Three valves dislodged, or an embolization occurred (one patient received a second valve while two patients required conversion to surgery), one patient had coronary obstruction (patient required urgent coronary artery bypass), two patients experienced annular rupture (one patient required conversion to rescue surgery while one patient was treated conservatively), one aortic arch dissection occurred and eight patients had a paravalvular leak of grade > 2 (five received a second TAVI after proper ballooning, three patients were converted to surgical AVR). In summary, seven TAVI patients required intraprocedural conversion to surgery in an emergency, and four of them (57.1%) died in the perioperative period. Operative Outcomes are listed in Table 2.

### 3.2. Postoperative Results

Early outcomes and postoperative complications are listed in Table 3. No differences in terms of 30-day mortality were found between the two groups (matched 30-day mortality: SuRD-AVR 6/346 patients, 1.73% versus TAVI 7/346 patients, 2.02%, *p*-value: 0.779), nor differences in terms of postoperative stroke/TIA events (matched stroke/TIA: SuRD-AVR 3/346 patients, 0.87% versus, TAVI 5/346 patients, 1.47%, *p*-value: 0.729). Postoperative acute renal failure (ARF) was similar between groups (matched ARF: SuRD-AVR 14/346 patients, 4.05% versus TAVI 9/346 patients, 2.60%, *p*-value: 0.289).

Postoperative need for blood transfusions was significantly lower in patients receiving TAVI (matched blood transfusions: SuRD-AVR, 51/346 patients 14.7% vs. TAVI 7/346 patients, 2.0%; *p* < 0.001), while the incidence of peripheral vascular complications was significantly higher in the TAVI group (matched vascular complications: SuRD-AVR 3/346 patients, 0.9% vs. TAVI 17/346 patients, 4.9%, *p* < 0.001). Finally, the incidence of permanent PM implantation (PPI) and PVL of grade ≥ 2 were significantly higher in TAVI group when compared to SuRD-AVR (matched PPM: SuRD-AVR 23/346, 6.7% vs. TAVI 38/346, 10.9%; *p* = 0.044; matched PVL grade ≥ 2: SuRD-AVR 4/346 patients, 1.2% vs. TAVI 14/346 patients, 4.1%; *p*:0.029).

### 3.3. Mid-Term Outcomes

**Figure 2:**
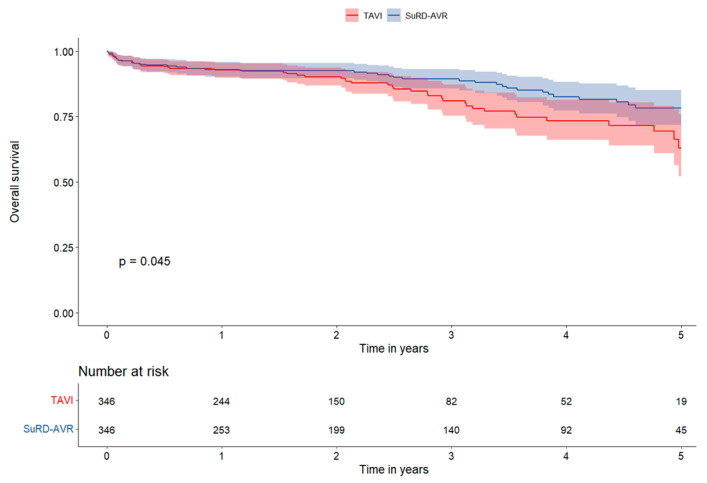
Survival Analysis Kaplan-Meier curves for all-cause death in the matched cohort.

**Figure 3:**
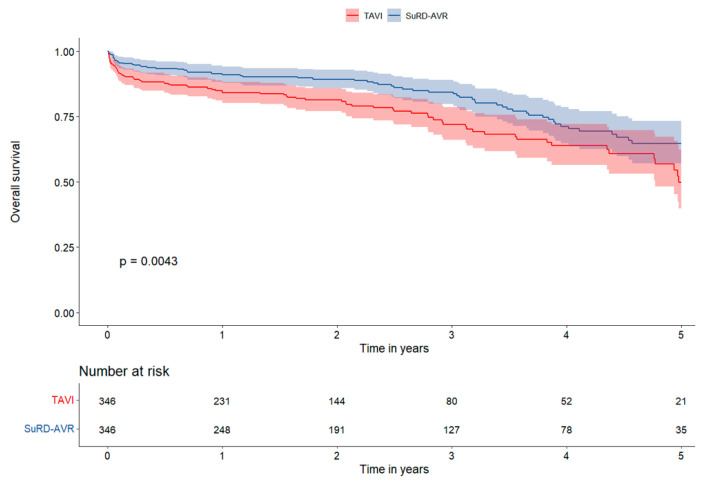
Survival Analysis Kaplan-Meier curves for survival freedom from MACCEs in the matched cohort.

The median follow-up time was 2.1 years (IQ range 0.9–3.7 years). The OS rate was higher in SuRD-AVR both in the matched and unmatched population after 5-year of follow-up (5-year matched: SuRD-AVR 78.5%, 95% CI 72.1–84.9% versus TAVI 62.9%, 95% CI 51.1–74.7%, *p* = 0.045). The 2, 3, and 4-year OS between matched SuRD-AVR and TAVI were 92.0% (95% CI: 89.0–95.1) vs. 89.6% (95% CI: 86.0–93.3), 88.6% (95% CI: 84.7–92.7) vs. 80.0% (95% CI: 74.0–86.4), and 81.6% (95% CI: 76.0–87.5) vs. 71.6% (95% CI: 63.9–80.2), respectively, (Figure 2). Patients in the SuRD-AVR group had higher survival freedom from MACCEs both in matched and unmatched populations at 5-years follow-up (5-years matched: SuRD-AVR 64.6%, 95% CI 56.6–72.6% versus TAVI 48.7%, 95% CI 36.9–60.5%, *p* = 0.004). The 2, 3, and 4-year survival freedom from MACCEs between matched SuRD-AVR and TAVI were 88.7% (95% CI: 85.2–92.4) vs. 80.8% (95% CI: 76.4–85.4), 83.6% (95% CI: 79.0–88.4) vs. 71.1% (95% CI: 64.9–77.8), and 70.3% (95% CI: 63.5–77.7) vs. 62.3% (95% CI: 54.7–70.9), respectively (Figure 3). At the landmark analysis, SuRD-AVR showed a significantly higher OS at 4 years in the matched group (*p* = 0.043) and at 3 years in the non-matched group (*p* = 0.002), compared to TAVI. MACCEs were significantly lower in the SuRD-AVR compared to TAVI at 3 years both in the matched (*p* = 0.001) and non-matched group (*p* < 0.001). Landmark analysis curves are depicted in Supplementary Figures S1–S4.

Cox regression analysis at follow-up showed that patients undergoing TAVI had a higher risk of death at follow-up (HR 1.53, 95% CI 1.02–2.03, *p* = 0.040). Moreover, TAVI itself was found to be an independent risk factor for MACCEs (HR 2.56, 95% CI 1.75–3.73, *p* < 0.001). Finally, PPI was found to be an independent predictor of mortality (HR 1.82, 95% CI 1.11–2.99, *p* < 0.001) (Table 4).

## 4. Discussion

The main findings of this study can be summarized as follows: (I) 30-day mortality and incidence of stroke/TIA between SuRD-AVR and TAVI were comparable in low-risk patients; (II) the incidence of PPI and PVL ≥ 2 was higher in patients undergoing TAVI; (III) at 5-year follow-up SuRD-AVR showed better overall survival and survival freedom from MACCEs compared to TAVI; (IV) post-procedural PPI and TAVI procedure negatively affected survival at follow-up.

In the last decade, improvements in technologies and the increase in physicians’ experience have led to a gradual decrease in in-hospital mortality following both TAVI and sAVR. The trend towards treating patients with lower risk profiles with transcatheter heart valves may have favored this improvement in overall TAVI results [21].

Large randomized trials (namely NOTION, PARTNER 3 and The Evolut Low Risk) compared sAVR versus TAVI in patients at low-risk, giving an important insight into this topic. However, concerns arise from the results of these studies. The NOTION Trial reported a 30-day mortality in the TAVI group (2.1%) consistent with ours and previous studies [7,8,9,22,23]. Surprisingly, the mortality in the surgical arm of NOTION was higher than expected (EuroSCORE II predicted mortality: 2.0% vs. Observed mortality: 3.7%). The mortality rate of the surgical population of the NOTION trial was higher even if compared to isolated sAVR outcomes reported in the 2021 STS Database (mortality: 1.9%) [24], raising concerns about patient selection. In the PARTNER 3 Trial, designed to randomize the same subset of low-risk patients, early mortality of sAVR versus TAVI was similar (sAVR: 1.1% vs. TAVI: 0.4%), but in both groups, mortality was very low when compared to NOTION early mortality [3,7]. Furthermore, in the PARTNER 3 trial, 26.4% of patients in the surgical arm received a concomitant surgical procedure that might have had a clinical impact, especially in terms of death, stroke and early repeated hospitalization [3,24,25]. Moreover, the PARTNER 3 trial demonstrated that the rate of the composite endpoint of death, stroke, or rehospitalization at 1 year was significantly lower in the TAVI group, but this difference was mainly influenced by the significantly higher rate of rehospitalization in the first 30 days, while no differences were reported in terms of death and stroke [3]. The OBSERVANT study reports, consistent with the results of these trials, having comparable early outcomes in terms of mortality and MACCEs between surgery and TAVI in patients at low-risk. This study, steady with our research and different from PARTNER 3, enrolled patients undergoing isolated aortic valve treatment, while the OBSERVANT study selected patients with a slightly higher risk profile and included only transfemoral TAVI patients [2].

To date, results about the use of SuRD-AVR compared to TAVI have not yet been evaluated in large RCTs, especially in low surgical risk patients. In this study, we analyzed for the first time the postoperative results and outcomes up to 5-years of sutureless and rapid deployment valves compared to TAVI in patients with isolated aortic stenosis at low surgical risk.

In our real-world analysis comparing sutureless and rapid deployment valves versus TAVI in low-risk patients, no significant differences were reported for hard endpoints such as mortality and stroke/TIA at 30 days. These results are supported by previous “real-world” propensity-matched studies by Rosato and Schaefers reporting no significant differences in 30-day mortality and stroke/TIA in low-risk patients when sAVR was compared to TAVI [8,9]. Similarly, a propensity-matched study from Vilalta showed no significant differences in terms of 30-day mortality and stroke/TIA between SuRD-AVR and TAVI, according to results from the GARY registry [22,23].

Consistent with our results, the PARTNER 3 trial, the Evolut Low Risk and NOTION trials [3,6,7] showed no differences in terms of mortality and stroke/TIA between sAVR and TAVI at 1-year follow-up. However, at 2-year follow-up, the Evolut Low Risk and PARTNER 3 trials depicted a tendency of increased adverse events rate (death, stroke and TIA) in the TAVI group, although not significant. These results indicate a higher mortality and stroke/TIA incidence between 1- and 2-year of follow-up in the TAVI group when compared to sAVR [26]. A similar trend was also observed between 2 and 5-year follow-up in the PARTNER 2 trial in patients with intermediate-risk profiles [1].

In this scenario, two meta-analyses, including PSM and RCTs, reported conflicting results regarding the comparison between transcatheter versus surgical aortic valve replacement in patients at low surgical risk [27,28]. Witberg et al. reported no differences in short-term mortality between TAVR or SAVR (2.2% for TAVR and 2.6% for SAVR, RR 0.89, 95% CI 0.56–1.41, *p* = 0.62), while TAVR was associated with increased risk for intermediate-term mortality (17.2% for TAVR and 12.7% for SAVR, RR 1.45, 95% CI 1.11–1.89, *p* = 0.006) [27]. On the other hand, Rawasia and colleagues reported lower short-term mortality for TAVI vs. SAVR (1.8% vs. 2.8%, RR = 0.67, [0.56–0.80]), while mid-term all-cause mortality was similar between TAVI and SAVR (8.6% vs. 8.4%, RR = 0.90 [0.66–1.24]), but was lower with TAVI in RCTs (2.1% vs. 3.5%, RR = 0.61 [0.39–0.95]).

To date, the NOTION trial is the only RCT reporting results beyond five years of follow-up showing comparable outcomes between sAVR and TAVI in low-risk patients [29]. However, the small proportion of randomized patients compared to the screened patients’ population limited the power of the study [7]. In addition, this trial showed two criticalities: (I) 40% of patients included in the surgical arm presented a small aortic annulus (19–21 mm), increasing the risk of patient-prosthesis mismatch, that represents an independent predictor of mortality [30]; (II) some of the bioprostheses used (i.e., Sorin Mitroflow 10%, St. Jude Trifecta 24%) have been associated with an increased incidence of degeneration at 5 years, poor hemodynamic performance and a negative impact on survival [31,32]. In our study, results beyond 3 years of follow-up showed a significantly lower survival in patients receiving TAVI when compared to SuRD-AVR, consistent with the results of Rosato and Schaefers [8,9]. Similar findings are reported in the latest update of the OBSERVANT study showing increased mortality and MACCE incidence in the TAVI group at five years; of note in this study, during the five year follow-up, a higher incidence of PPI was reported in the TAVI group [2]. A possible explanation of these findings could be represented by the higher incidence of PPI as well as PVL ≥ 2, which occurred in TAVI patients (PVL ≥ 2: SuRD-AVR 1.2% vs. TAVI 4.1%, *p* = 0.029; PPI: SuRD-AVR 6.7% vs. TAVI 10.9%, *p* = 0.044). Paravalvular regurgitation, as well as permanent pacemaker implantation, have been demonstrated to represent independent predictors of mortality in patients at intermediate/high surgical risk [10,11,12,13], and in our study, we found similar associations between PPI and 5-year mortality (HR 1.82, 95% CI 1.11–2.99, *p* < 0.001).

In this study, we reported a significantly lower incidence of moderate-to-severe PVL in the SuRD-AVR group, according to the results of the GARY registry [23]. While the incidence of PVL in our surgical arm was consistent with previous data on sutureless and rapid deployment valves [22,23], we found a slightly higher incidence of moderate-to-severe PVL in the TAVI group (4.1%) compared to other studies (PARTNER 3: 1.1%, Evolut Low Risk 3.4%) [3,6]. This could be due to the inclusion of first-generation devices without outer sealing skirts in the TAVI group, which may have jeopardized the effective incidence of PVL, similar to the low-risk observational study by Schaefer and colleagues [8]. Cox regression analyses could not demonstrate any association between PVL ≥ 2 and mortality: since the overall incidence of significant PVL rate has reduced over time in TAVI procedures, the impact of this complication on survival could now be less evident. On the other hand, other studies also suggested that mild PVL has a negative prognostic role [33]. Further studies about the impact of mild PVL and its worsening at follow-up are needed to clarify its impact on survival in low-risk patients.

### Study Limitations

The present study is a non-randomized observational study. The lack of randomization may lead to some selection biases. The propensity score matching methodology may eliminate most of these biases, but some residual confounding factors could persist. In particular, TAVI patients may suffer from comorbidities not taken into account by EuroSCORE II. Illnesses such as porcelain aorta, malignancies, increased calcium load of the cardiovascular system, status post chest irradiation or hostile chest may lead to ineligibility for surgical AVR. This emphasizes again the difficulty of defining a ‘real’ low-risk patient with current risk stratification tools.

## 5. Conclusions

This “real-world” propensity-matched study compared low surgical risk patients with isolated aortic stenosis treated with TAVI or sutureless and rapid deployment aortic valves. We found that SuRD-AVR was associated with improved outcomes at follow-up in terms of primary (5-year all-cause death) and secondary endpoint (survival freedom from MACCEs). Multivariate Cox regression analysis identified permanent pacemaker implantation (PPI) and TAVI as independent predictors of mortality in this low-risk population.

## Figures and Tables

**Table 1:** Preoperative characteristics.

	UNMATCHED			MATCHED		
	SuRD-AVR (n = 636)	TAVI (n = 670)	*p*-Value	SuRD-AVR (n = 346)	TAVI (n = 346)	*p*-Value
Age (years), median (IQR)	78 (74–81)	81 (77–84)	<0.001	79 (76–83)	80 (76–84)	0.266
Gender (female)	364 (57.2%)	312 (46.6%)	<0.001	186 (53.8%)	189 (54.6%)	0.819
Body mass index (kg/m^2^)	27.1 ± 4.6	27.2 ± 4.3	0.642	26.9 ± 4.7	27.2 ± 4.9	0.069
Body surface area (m^2^), median (IQR)	1.81 ± 0.2	1.82 ± 0.2	0.773	1.81 ± 0.2	1.81 ± 0.2	0.923
EuroSCORE II (%), median (IQR)	2.0 (1.4–2.8)	3.0 (2.3–3.5)	<0.001	2.6 (1.9–3.2)	2.5 (2.0–3.1)	0.977
Hypertension(mmHg)	532 (83.6%)	566 (84.5%)	0.682	282 (81.5%)	291 (84.1%)	0.365
Dyslipidaemia	428 (67.3%)	359 (53.6%)	<0.001	196 (56.6%)	195 (56.4%)	0.939
Diabetes Mellitus	208 (32.7%)	141 (21.0%)	<0.001	92 (26.6%)	83 (24.0%)	0.431
Advance NYHA (class III–IV)	253 (39.8%)	257 (38.4%)	0.598	79 (22.8%)	82 (23.7%)	0.787
COPD (FEV1 < 60%)	69 (10.8%)	93 (13.9%)	0.097	46 (13.3%)	49 (14.2%)	0.740
Previous CVA	50 (7.9%)	45 (6.7%)	0.426	21 (6.1%)	16 (4.6%)	0.398
Atrial fibrillation	63 (9.9%)	63 (9.4%)	0.758	36 (10.4%)	30 (8.7%)	0.437
Pulmonary hypertension (>30 mmHg)	179 (28.1%)	228 (34%)	0.022	114 (32.9%)	112 (32.4%)	0.871
Peripherical arterial disease	68 (10.7%)	67 (10%)	0.681	35 (10.1%)	31 (9.0%)	0.604
Previous Cardiac Surgery	12 (1.9%)	152 (22.7%)	<0.001	12 (3.5%)	17 (4.9%)	0.343
Frailty	19 (2.9%)	33 (4.9%)	0.073	10 (2.8%)	15 (4.3%)	0.308
Creatinine (mg/dL), mean ± SD	0.95 ± 0.5	1.15 ± 0.6	<0.001	1.01 ± 0.7	1.06 ± 0.6	0.324
Sievers Type 1 Bicupid valve	44 (6.7%)	7 (1%)	<0.001	6 (1.7%)	5 (1.4%)	0.761
SAA	111 (17.4%)	139 (20.7%)	0.130	67 (19.3%)	86 (24.8%)	0.081
Left ventricular ejection fraction, mean ± SD	57.7 ± 6	57.7 ± 7	0.478	57.6 ± 6	56.4 ± 7	0.231

NYHA: New York Heart Association; COPD: Chronic Obstructive Pulmonary Disease; SAA: Small Aortic Annulus.

**Table 2:** Operative results.

	UNMATCHED			MATCHED		
	SuRD-AVR (n = 636)	TAVI (n = 670)	*p*-Value	SuRD-AVR (n = 346)	TAVI (n = 346)	*p*-Value
Sternotomy, n (%)	230 (36.2%)			141 (40.8%)		
MICS, n (%)	406 (63.8%)			205 (59.2%)		
CPB time(min), median (IQR)	62 (51–83)			63 (49–84)		
Aortic cross-clamp time (min), median (IQR)	42 (32–55)			39 (30–54)		
TF-TAVI, n (%)		569 (84.9%)			304 (87.9%)	
TV-TAVI, n (%)		87 (13%)			34 (9.8%)	
TA-TAVI, n (%)		14 (2.1%)			8 (2.3%)	
Valve size (mm), median (IQR)	23 (23–25)	26 (23–26)	**<0.001**	23 (23–25)	26 (23–26)	**<0.001**
Non-elective procedure, n (%)	6 (0.94%)	7 (1.04%)	0.853	3 (0.86%)	5 (1.45%)	0.721
ICU stay(days), median (IQR)	1 (0–1.5)	1 (0–2)	0.258	1 (0–2)	1 (0–2)	0.672
ECHO at discharge						
Postoperative EF%	50 (49–60)	50 (50–55)	0.653	50 (50–60)	50 (47–56)	0.583
Mean transvalvular gradient (mmHg), mean ± SD	10.2 ± 5.1	10.7 ± 4.7	0.452	10.6 ± 4.8	10.8 ± 5.1	0.765
EOA (cm2), mean ± SD	1.7 ± 0.4	1.6 ± 0.5	0.379	1.8 ± 0.4	1.8 ± 0.5	0.830
PVL > 2	7 (1.1%)	23 (3.4%)	**0.005**	4 (1.2%)	14 (4.1%)	**0.029**

MICS: Minimally Invasive Cardiac Surgery; TF: Transfemoral; TV: Transvessel; TA:Transapical. Statistically significant *p*-values (*p* < 0.05) are highlighted in bold.

**Table 3:** Postoperative results.

	UNMATCHED			MATCHED		
	Surgery (n = 636)	TAVR (n = 670)	*p*-Value	Surgery (n = 346)	TAVR (n = 346)	*p*-Value
Procedural Mortality	2 (0.31%)	4 (0.60%)	0.450	1 (0.29%)	1 (0.29%)	1.000
30-Days Mortality	13 (2.04%)	19 (2.84%)	0.355	6 (1.73%)	7 (2.02%)	0.779
Cardiac Death	5 (0.9%)	8 (1.2%)	0.837	3 (0.87%)	4 (1.16%)	1.000
PPI	51 (8.0%)	95 (14.2%)	**<0.001**	23 (6.7%)	38 (10.9%)	**0.044**
Surgical revision for bleeding	28 (4.4%)	6 (0.9%)	**<0.001**	21 (6.1%)	2 (0.6%)	**<0.001**
Blood Transfusion	93 (14.6%)	25 (3.7%)	**<0.001**	51 (14.7%)	7 (2.0%)	**<0.001**
Atrial Fibrillation	44 (6.9%)	167 (24.9%)	**<0.001**	21 (6.0%)	76 (21.9%)	**<0.001**
Acute renal failure	26 (4.08%)	17 (2.54%)	0.117	14 (4.05%)	9 (2.60%)	0.289
Stroke	4 (0.63%)	9 (1.34%)	0.267	3 (0.87%)	5 (1.47%)	0.725
Endocarditis	3 (0.47%)	4 (0.60%)	1	2 (0.7%)	3 (0.9%)	0.784
Vascular complication	4 (0.63%)	29 (4.33%)	**0.001**	3 (0.9%)	17 (4.9%)	**0.001**

PPI: Permanent pacemaker Implant. Statistically significant *p*-values (*p* < 0.05) are highlighted in bold.

**Table 4:** Univariate and Multivariate Cox Regression analysis for all-cause death.

	UNIVARIABLE			MULTIVARIABLE		
	HR	95% CI	*p*-Value	HR	95% CI	*p*-Value
PPI (TVC)	1.87	1.14–3.06	**0.012**	1.82	1.11–2.99	**<0.001**
PVL (TVC)	0.29	0.04–2.14	0.230			
SAA	1.06	0.64–1.73	0.814			
TAVI vs. SuRD-AVR	1.53	1.02–2.03	**0.040**	1.74	1.12–2.71	**0.013**
PAPs > 30 mmHg	1.06	0.69–1.64	0.771			
LVEF < 35%	0.81	0.19–3.35	0.780			
III stage CKD	3.39	1.67–6.89	<0.001	3.45	1.70–7.01	<0.001

PPI: Permanent Pacemaker Implantation, TVC: Time Varying Covariate; SAA: Small Aortic Annulus echocardiographic annulus < 21 mm; PAP: Pulmonary Artery Pressure; LVEF: Left Ventricular Ejection fraction; CKD: Chronic Kidney Disease. Statistically significant *p*-values (*p* < 0.05) are highlighted in bold.

## Data Availability

The data underlying this article will be shared on reasonable request to the corresponding author.

## Supplementary Materials

The following supporting information can be downloaded at: https://www.mdpi.com/article/10.3390/jcm12124045/s1. Supplementary Figure S1—Landmark analysis for overall survival in the matched group; Supplementary Figure S2—Landmark analysis for overall survival in the non-matched group; Supplementary Figure S3—Landmark analysis for survival freedom from MACCE in the matched group; Supplementary Figure S4—Landmark analysis for survival freedom from MACCE in the non-matched group.

## References

[1] Makkar R.R., Thourani V.H., Mack M.J., Kodali S.K., Kapadia S., Webb J.G., Yoon S.-H., Trento A., Svensson L.G., Herrmann H.C. (2020). Five-Year Outcomes of Transcatheter or Surgical Aortic-Valve Replacement. N. Engl. J. Med..

[2] Barbanti M., Tamburino C., D’errigo P., Biancari F., Ranucci M., Rosato S., Santoro G., Fusco D., Seccareccia F. (2019). Five-Year Outcomes of Transfemoral Transcatheter Aortic Valve Replacement or Surgical Aortic Valve Replacement in a Real World Population. Circ. Cardiovasc. Interv..

[3] Mack M.J., Leon M.B., Thourani V.H., Makkar R., Kodali S.K., Russo M., Kapadia S.R., Malaisrie S.C., Cohen D.J., Pibarot P. (2019). Transcatheter Aortic-Valve Replacement with a Balloon-Expandable Valve in Low-Risk Patients. N. Engl. J. Med..

[4] Vahanian A., Beyersdorf F., Praz F., Milojevic M., Baldus S., Bauersachs J., Capodanno D., Conradi L., De Bonis M., De Paulis R. (2022). 2021 ESC/EACTS Guidelines for the management of valvular heart disease. Eur. Heart J..

[5] Otto C.M., Nishimura R.A., Bonow R.O., Carabello B.A., Erwin J.P., Gentile F., Jneid H., Krieger E.V., Mack M., McLeod C. (2021). 2020 ACC/AHA Guideline for the Management of Patients with Valvular Heart Disease: Executive Summary: A Report of the American College of Cardiology/American Heart Association Joint Committee on Clinical Practice Guidelines. Circulation.

[6] Popma J.J., Deeb G.M., Yakubov S.J., Mumtaz M., Gada H., O’Hair D., Bajwa T., Heiser J.C., Merhi W., Kleiman N.S. (2019). Transcatheter Aortic-Valve Replacement with a Self-Expanding Valve in Low-Risk Patients. N. Engl. J. Med..

[7] Thyregod H.G.H., Steinbrüchel D.A., Ihlemann N., Nissen H., Kjeldsen B.J., Petursson P., Chang Y., Franzen O.W., Engstrøm T., Clemmensen P. (2015). Transcatheter Versus Surgical Aortic Valve Replacement in Patients with Severe Aortic Valve Stenosis: 1-Year Results from the All-Comers NOTION Randomized Clinical Trial. J. Am. Coll. Cardiol..

[8] Schaefer A., Schofer N., Goßling A., Seiffert M., Schirmer J., Deuschl F., Schneeberger Y., Voigtländer L., Detter C., Schaefer U. (2019). Transcatheter aortic valve implantation versus surgical aortic valve replacement in low-risk patients: A propensity score-matched analysis. Eur. J. Cardiothorac. Surg..

[9] Rosato S., Santini F., Barbanti M., Biancari F., D’errigo P., Onorati F., Tamburino C., Ranucci M., Covello R.D., Santoro G. (2016). Transcatheter Aortic Valve Implantation Compared with Surgical Aortic Valve Replacement in Low-Risk Patients. Circ. Cardiovasc. Interv..

[10] Leon M.B., Smith C.R., Mack M.J., Makkar R.R., Svensson L.G., Kodali S.K., Thourani V.H., Tuzcu E.M., Miller D.C., Herrmann H.C. (2016). Transcatheter or Surgical Aortic-Valve Replacement in Intermediate-Risk Patients. N. Engl. J. Med..

[11] Nazif T.M., Dizon J.M., Hahn R.T., Xu K., Babaliaros V., Douglas P.S., El-Chami M.F., Herrmann H.C., Mack M., Makkar R.R. (2015). Predictors and clinical outcomes of permanent pacemaker implantation after transcatheter aortic valve replacement: The PARTNER (Placement of AoRtic TraNscathetER Valves) trial and registry. JACC Cardiovasc. Interv..

[12] Arnold S.V., Manandhar P., Vemulapalli S., Kosinski A., Desai N.D., Bavaria J.E., Carroll J.D., Mack M.J., Thourani V.H., Cohen D.J. (2021). Impact of short-term complications of transcatheter aortic valve replacement on longer-term outcomes: Results from the STS/ACC Transcatheter Valve Therapy Registry. Eur. Heart J. Qual. Care Clin. Outcomes.

[13] Fischlein T., Folliguet T., Meuris B., Shrestha M.L., Roselli E.E., McGlothlin A., Kappert U., Pfeiffer S., Corbi P., Lorusso R. (2021). Sutureless versus conventional bioprostheses for aortic valve replacement in severe symptomatic aortic valve stenosis. J. Thorac. Cardiovasc. Surg..

[14] Andreas M., Wallner S., Habertheuer A., Rath C., Schauperl M., Binder T., Beitzke D., Rosenhek R., Loewe C., Wiedemann D. (2016). Conventional versus rapid-deployment aortic valve replacement: A single-centre comparison between the Edwards Magna valve and its rapid-deployment successor. Interact. Cardiovasc. Thorac. Surg..

[15] Fischlein T., Caporali E., Folliguet T., Kappert U., Meuris B., Shrestha M.L., Roselli E.E., Bonaros N., Fabre O., Corbi P. (2022). Randomized controlled trial between conventional versus sutureless bioprostheses for aortic valve replacement: Impact of mini and full sternotomy access at 1-year follow-up. Int. J. Cardiol..

[16] Sá M.P., Jabagi H., Dokollari A., Awad A.K., Eynde J.V.D., Malin J.H., Sicouri S., Torregrossa G., Ruhparwar A., Weymann A. (2022). Early and late outcomes of surgical aortic valve replacement with sutureless and rapid-deployment valves versus transcatheter aortic valve implantation: Meta-analysis with reconstructed time-to-event data of matched studies. Catheter. Cardiovasc. Interv..

[17] Glauber M., Miceli A., di Bacco L. (2020). Sutureless and rapid deployment valves: Implantation technique from A to Z—The Perceval valve. Ann. Cardiothorac. Surg..

[18] Glauber M., Miceli A., di Bacco L. (2020). Sutureless and rapid deployment valves: Implantation technique from A to Z—The INTUITY Elite valve. Ann. Cardiothorac. Surg..

[19] Capodanno D., Petronio A.S., Prendergast B., Eltchaninoff H., Vahanian A., Modine T., Lancellotti P., Sondergaard L., Ludman P.F., Tamburino C. (2017). Standardized definitions of structural deterioration and valve failure in assessing long-term durability of transcatheter and surgical aortic bioprosthetic valves: A consensus statement from the European Association of Percutaneous Cardiovascular Interventions (EAPCI) endorsed by the European Society of Cardiology (ESC) and the European Association for Cardio-Thoracic Surgery (EACTS). Eur. J. Cardiothorac. Surg..

[20] Généreux P., Piazza N., Alu M.C., Nazif T., Hahn R.T., Pibarot P., Bax J.J., Leipsic J.A., Blanke P., Blackstone E.H. (2021). Valve Academic Research Consortium 3: Updated Endpoint Definitions for Aortic Valve Clinical Research. J. Am. Coll. Cardiol..

[21] Gaede L., Blumenstein J., Eckel C., Grothusen C., Tiyerili V., Sötemann D., Nef H., Elsässer A., Achenbach S., Möllmann H. (2022). Transcatheter-based aortic valve replacement vs. isolated surgical aortic valve replacement in 2020. Clin. Res. Cardiol..

[22] Vilalta V., Alperi A., Cediel G., Mohammadi S., Fernández-Nofrerias E., Kalvrouziotis D., Delarochellière R., Paradis J.-M., González-Lopera M., Fadeuilhe E. (2021). Midterm Outcomes Following Sutureless and Transcatheter Aortic Valve Replacement in Low-Risk Patients with Aortic Stenosis. Circ. Cardiovasc. Interv..

[23] Abdel-Wahab M., Fujita B., Frerker C., Bauer T., Beckmann A., Bekeredjian R., Bleiziffer S., Möllmann H., Walther T., Hamm C. (2020). Transcatheter Versus Rapid-Deployment Aortic Valve Replacement: A Propensity-Matched Analysis from the German Aortic Valve Registry. JACC Cardiovasc. Interv..

[24] Bowdish M.E., D’agostino R.S., Thourani V.H., Schwann T.A., Krohn C., Desai N., Shahian D.M., Fernandez F.G., Badhwar V. (2021). STS Adult Cardiac Surgery Database: 2021 Update on Outcomes, Quality, and Research. Ann. Thorac. Surg..

[25] Alperi A., Voisine P., Kalavrouziotis D., Dumont E., Dagenais F., Perron J., Silva I., Bernardi F., Mohammadi S., Rodés-Cabau J. (2021). Aortic Valve Replacement in Low-Risk Patients with Severe Aortic Stenosis Outside Randomized Trials. J. Am. Coll. Cardiol..

[26] Leon M.B., Mack M.J., Hahn R.T., Thourani V.H., Makkar R., Kodali S.K., Alu M.C., Madhavan M.V., Chau K.H., Russo M. (2021). Outcomes 2 Years after Transcatheter Aortic Valve Replacement in Patients at Low Surgical Risk. J. Am. Coll. Cardiol..

[27] Witberg G., Lador A., Yahav D., Kornowski R. (2018). Transcatheter versus surgical aortic valve replacement inpatients at low surgical risk: A meta-analysis of randomized trials and propensity score matched observational studies. Catheter. Cardiovasc. Interv..

[28] Rawasia W.F., Usman M., Mujeeb F.A., Zafar M., Khan S.U., Alkhouli M. (2020). Transcatheter Versus Surgical Aortic Valve Replacement in Low-Surgical-Risk Patients: A Meta-Analysis of Randomized-Controlled Trials and Propensity-Matched Studies. Cardiovasc. Revasc. Med..

[29] Jørgensen T.H., Thyregod H.G.H., Ihlemann N., Nissen H., Petursson P., Kjeldsen B.J., Steinbrüchel D.A., Olsen P.S., Søndergaard L. (2021). Eight-year outcomes for patients with aortic valve stenosis at low surgical risk randomized to transcatheter vs. surgical aortic valve replacement. Eur. Heart J..

[30] Urso S., Sadaba R., Nogales E., Portela F. (2022). Why Does the NOTION Trial Show Poorer than Expected Outcomes in the Surgical Arm?. Hearts.

[31] Axtell A.L., Chang D.C., Melnitchouk S., Jassar A.S., Tolis G., Villavicencio M.A., Sundt T.M., D’Alessandro D.A. (2018). Early structural valve deterioration and reoperation associated with the mitroflow aortic valve. J. Card. Surg..

[32] Werner P., Coti I., Kaider A., Gritsch J., Mach M., Kocher A., Laufer G., Andreas M. (2022). Long-term durability after surgical aortic valve replacement with the Trifecta and the Intuity valve—A comparative analysis. Eur. J. Cardiothorac. Surg..

[33] Okuno T., Tomii D., Heg D., Lanz J., Praz F., Stortecky S., Reineke D., Windecker S., Pilgrim T. (2022). Five-year outcomes of mild paravalvular regurgitation after transcatheter aortic valve implantation. EuroIntervention.

