# Supplementary material for: Sutureless and Rapid Deployment vs. Transcatheter Valves for Aortic Stenosis in Low-Risk Patients: Mid-Term Results
Source: J Clin Med. 2023 Jun 14;12(12):4045. doi: 10.3390/jcm12124045 (PMC10299284; doi:10.3390/jcm12124045)

Supplementary Figure 1 – Landmark analysis for overall survival in the matched group

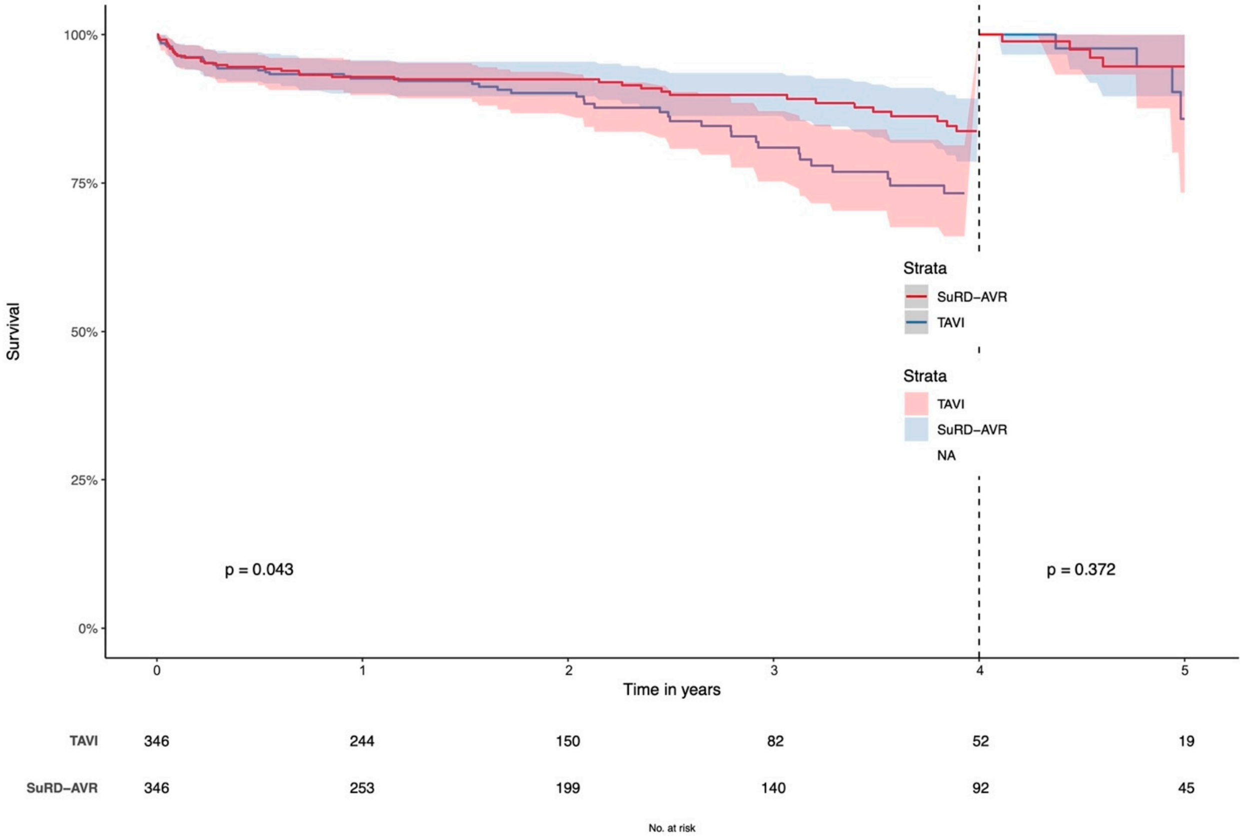

Supplementary Figure 2 - Landmark analysis for overall survival in the non-matched group

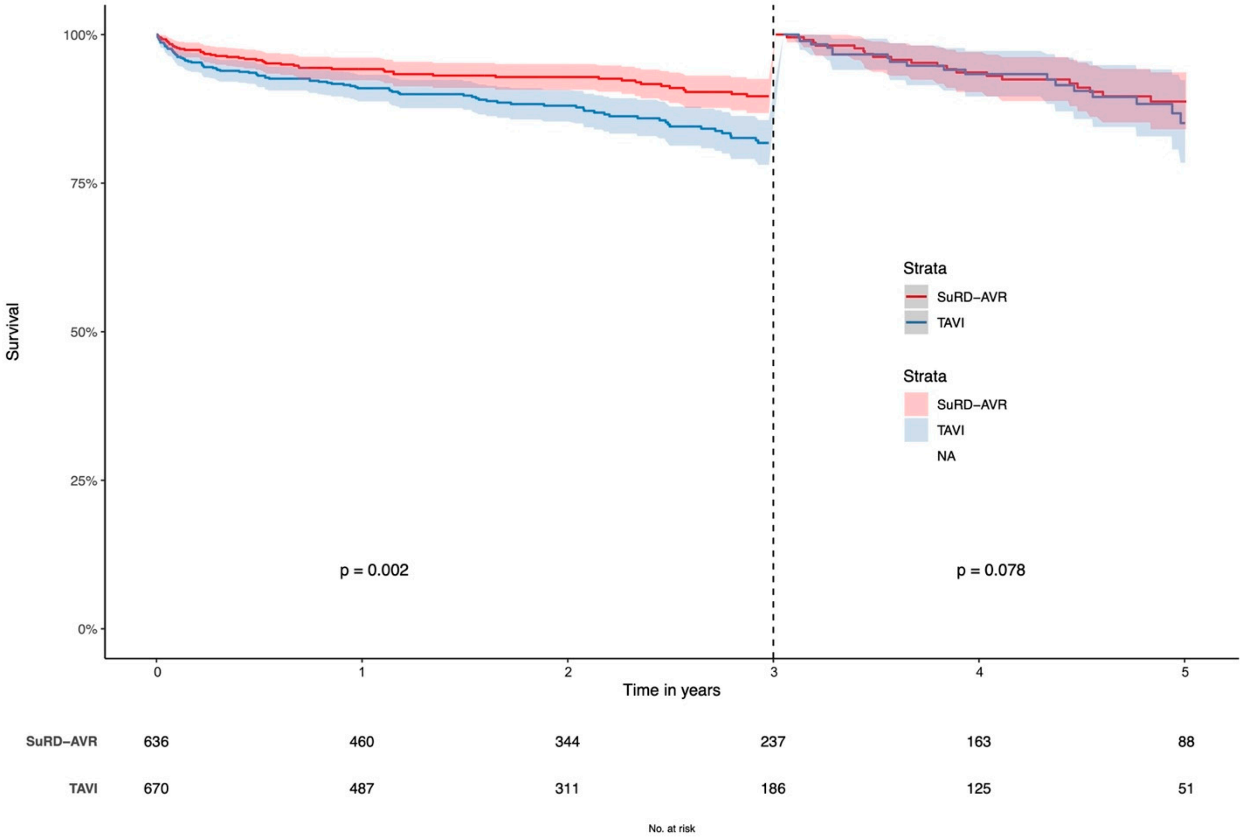

Supplementary Figure 3 - Landmark analysis for survival freedom from MACCE in the matched group

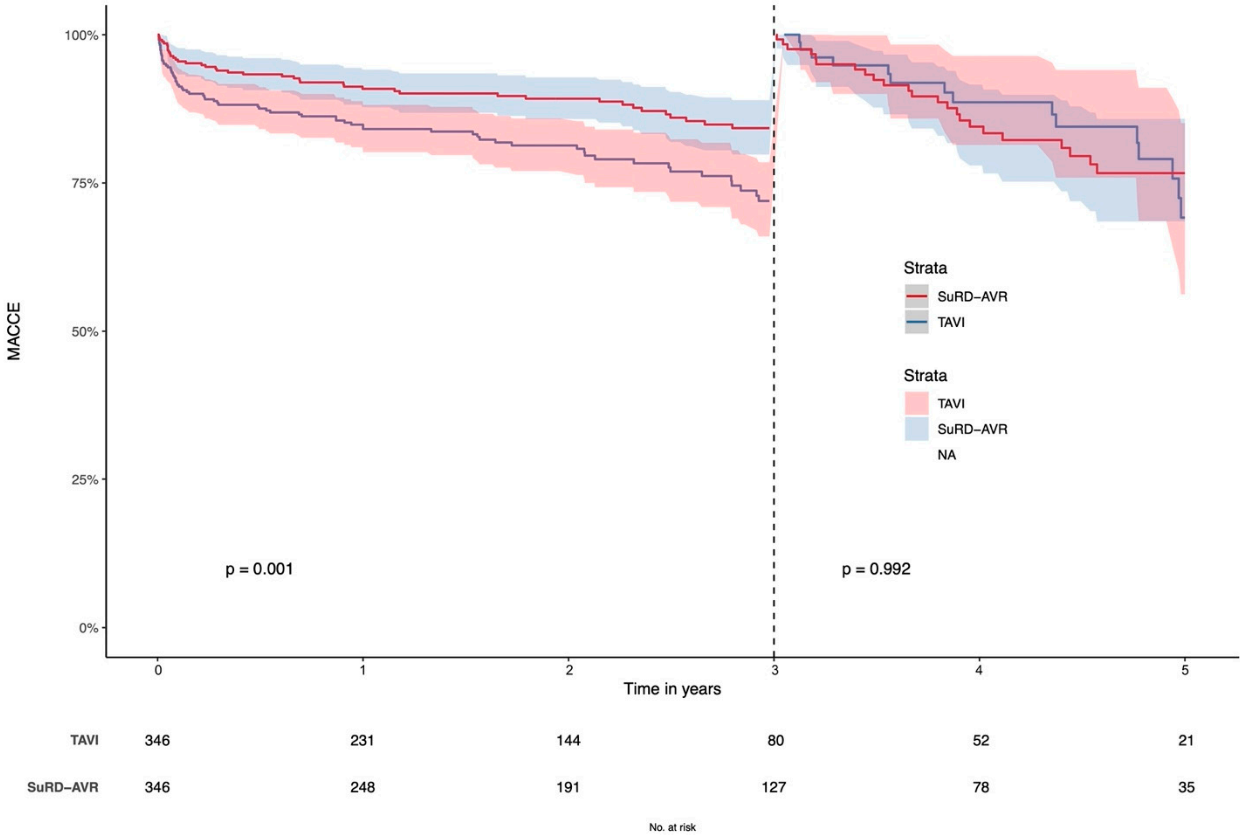

Supplementary Figure 4 - Landmark analysis for survival freedom from MACCE in the non-matched group

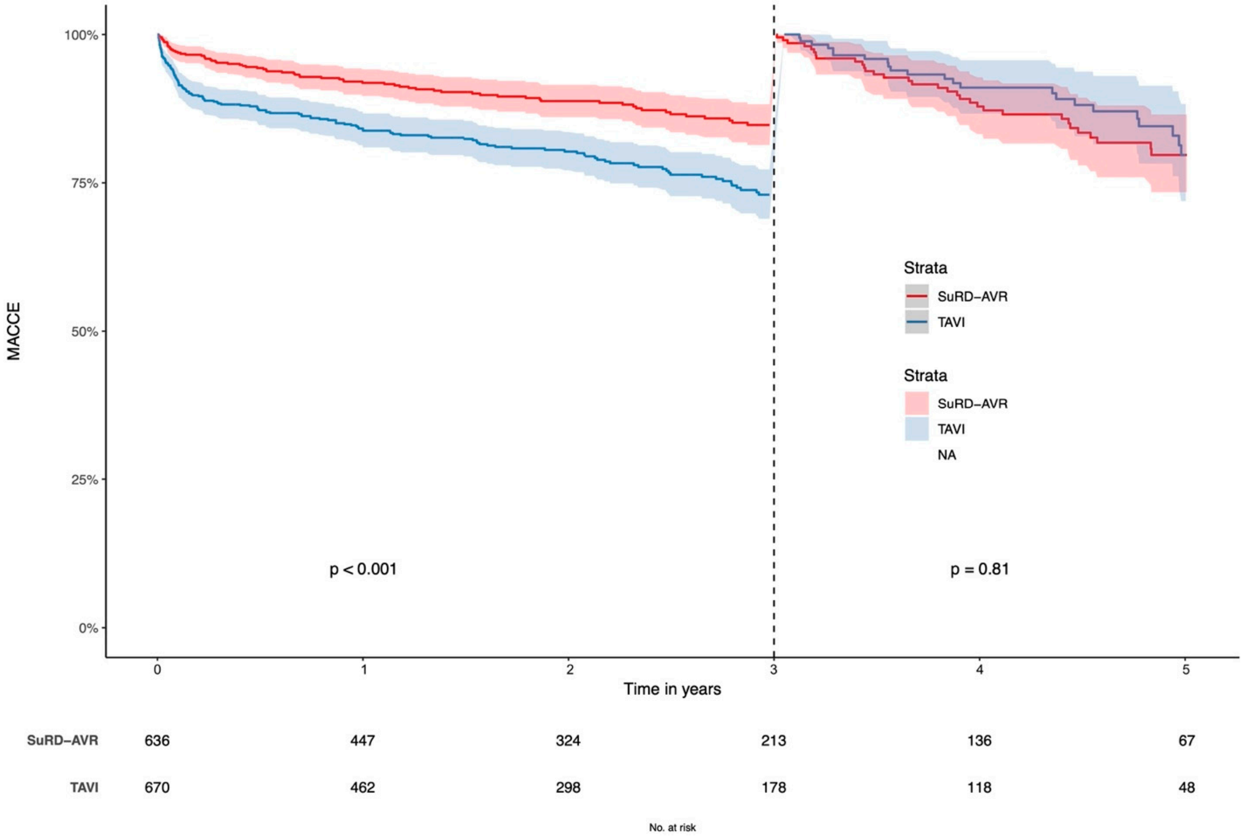

Supplement: Supplementary file 1 [file jcm-12-04045-s001.zip › jcm-2395893-supplementary.pdf]
